# Pattern-driven neighborhood search for biclustering of microarray data

**DOI:** 10.1186/1471-2105-13-S7-S11

**Published:** 2012-05-08

**Authors:** Wassim Ayadi, Mourad Elloumi, Jin-Kao Hao

**Affiliations:** 1 LERIA, Université d'Angers, 2 Boulevard Lavoisier, 49045 Angers Cedex 01, France; 2 LaTICE, Higher School of Sciences and Technologies of Tunis, 5 Avenue Taha Hussein, B. P. : 56, Bab Menara, 1008 Tunis, University of Tunis, Tunisia

## Abstract

**Background:**

Biclustering aims at finding subgroups of genes that show highly correlated behaviors across a subgroup of conditions. Biclustering is a very useful tool for mining microarray data and has various practical applications. From a computational point of view, biclustering is a highly combinatorial search problem and can be solved with optimization methods.

**Results:**

We describe a stochastic pattern-driven neighborhood search algorithm for the biclustering problem. Starting from an initial bicluster, the proposed method improves progressively the quality of the bicluster by adjusting some genes and conditions. The adjustments are based on the quality of each gene and condition with respect to the bicluster and the initial data matrix. The performance of the method was evaluated on two well-known microarray datasets (*Yeast cell cycle *and *Saccharomyces cerevisiae*), showing that it is able to obtain statistically and biologically significant biclusters. The proposed method was also compared with six reference methods from the literature.

**Conclusions:**

The proposed method is computationally fast and can be applied to discover significant biclusters. It can also used to effectively improve the quality of existing biclusters provided by other biclustering methods.

## Background

The DNA microarray technology permits to monitor and to measure gene expression levels for 10s of 1000s of genes simultaneously in a cell mixture in a single experiment under diverse experimental conditions. DNA microarray data are typically represented by a large matrix where each row contains the gene expression levels under specific conditions (columns). Since its invention, this technology has found many applications in biological and medical research. For instance, it is being used in cancer studies to better understand the biological mechanisms underlying oncogenesis, to discover new targets and new drugs, and to develop predictors for tailoring individualized treatments [1-3].

Microarray data analysis is a critical step in practical applications and often achieved with the help of data mining techniques [4]. Microarray data analysis can be performed according to at least two different and complementary approaches [1-3]. The first approach is based on supervised classification (also called class prediction or class discrimination). This generally involves selecting predictive genes to build a classifier that can be used to predict the outcome of new samples based on their expression profiles. Various methods based on this approach have been proposed in the literature and examples can be found in [5-15].

Another general approach for microarray data analysis relies on non-supervised classification (or clustering) methods. These cluster analysis methods try to identify groups of genes, or/and groups of conditions (samples), that exhibit similar expression patterns [16-20]. In the context of cluster analysis, biclustering is a particularly interesting approach which aims to identify simultaneously groups of genes and conditions (called biclusters) such that the genes of a bicluster show similar expression patterns across the selected conditions [21-23]. Formally, given a gene expression data matrix *M*(*I, J*) with gene index *i *∈ *I*={1, 2,..., *n*} and condition index *j *∈ *J*={1, 2,..., *m*} (*n *>>*m*), a bicluster *M*(*I', J'*) is a group of genes associated with a group of conditions such that *I' *⊆ *I *and *J' *⊆*J*. This paper focuses on finding meaningful biclusters for a given microarray dataset.

From a computational point of view, the biclustering problem is a highly combinatorial search problem and known to be NP-hard [22,24]. A number of heuristic search algorithms have been proposed and some recent reviews can be found in [22,25,26]. Generally, existing biclustering algorithms belong to one of the following approaches.

1. Greedy iterative search approach: Greedy biclustering algorithms build a solution by starting from the initial data matrix (or a transformed matrix) and iteratively remove bad genes/conditions according to a quality criterion. For instance, the algorithm presented in [27] (called Maximum Similarity Biclusters) starts by constructing a similarity matrix based on a reference gene. A greedy strategy is then iteratively applied to remove genes/conditions such that a maximum similarity is achieved in the remaining matrix (bicluster). Greedy algorithms can also proceed by extending greedily an initially empty bicluster. Examples of greedy biclustering algorithms can be found in [27-30]. They differ essentially in the way genes/conditions are added/removed. Greedy algorithms are computationally fast, but the quality of the biclusters found may be mediocre.

2. Biclusters enumeration approach: This approach tries to enumerate (implicitly) all the biclusters. The enumeration process is often represented by a search tree. During the construction of the search tree, some nodes are closed as soon as some pruning conditions are fulfilled. For instance, in [31], the authors propose the CE-Tree algorithm which builds its tree of biclusters by applying a special local breadth-first within a global depth-first search strategy in combination of exploring Maximum Dimension Sets for each pair of conditions. Representative examples of algorithms adopting this enumeration approach are given in [23,29,32-34]. This approach has the advantage of achieving high quality solutions. However, algorithms using this approach are expensive in computing time and memory space.

3. Stochastic search approach: This approach can be further divided into neighborhood search and evolutionary search. For neighborhood search, one begins with an initial candidate solution (bicluster) and improves iteratively its quality by replacing the bicluster with a neighboring bicluster. The neighboring bicluster is typically obtained by replacing a gene/condition by a better one. Cheng and Church [24] are probably the first to apply this approach to the biclustering problem. They employ the *Mean Squared Residue *(MSR) to measure the goodness of genes and conditions and to decide which genes/conditions are to be removed/added. Other biclustering algorithms based on local search are presented in [24,35-38]. Population-based evolutionary search generalizes neighborhood search by operating on a pool of candidate solutions. Candidate solutions are improved with operators like crossover and mutation. Examples of evolutionary biclustering algorithms can be found in [39-42].

In this paper we introduce a stochastic neighborhood search algorithm called *Pattern-Driven Neighborhood Search *(PDNS) for the biclustering problem. PDNS is based on a solution representation encoded as a behavior matrix and a dedicated neighborhood taking into account various patterns information. It also employs fast greedy algorithms to generate diversified initial biclusters of reasonable quality and a randomized perturbation strategy.

## Method

### Preprocessing of gene expression matrix

Prior to the search by PDNS, our method first applies a preprocessing step to transform the input data matrix *M *to a Behavior Matrix *M'*. This preprocessing step aims to highlight the trajectory patterns of genes. Indeed, according to [43-45], in microarray data analysis, genes are considered to be in the same cluster if their trajectory patterns of expression levels are similar across a set of conditions. Within the transformed matrix *M'*, each row represents the trajectory pattern of a gene across all the combined conditions while each column represents the trajectory pattern of all the genes under a pair of particular conditions in the data matrix *M*. The whole matrix *M' *provides thus useful information for the identification of relevant biclusters and the definition of a meaningful neighborhood of a local search algorithm.

Formally, the behavior matrix *M' *is constructed progressively by merging a pair of columns (conditions) from the input data matrix *M*. Since *M *has *n *rows and *m *columns, there is *m*(*m*−1)/2 distinct combinations between columns, represented by *J''*. So, *M' *has *n *rows and *m*(*m*−1)/2 columns. *M' *is defined as follows:

(1) $$M^{\prime }\left[i,l\right]=\left\{\begin{array}{c} 1\;if\;M\left[i,k\right]<M\left[i,q\right]\\ 0\;if\;M\left[i,k\right]=M\left[i,q\right]\\ -1\;if\;M\left[i,k\right]>M\left[i,q\right] \end{array}\right)$$

with *i *∈ [1..*n*], *l *∈ [1..*J''*], *k *∈ [1..*m*- 1], *q *∈ [2..*m*] and *q *≥ *k *+ 1.

**Figure 1:**
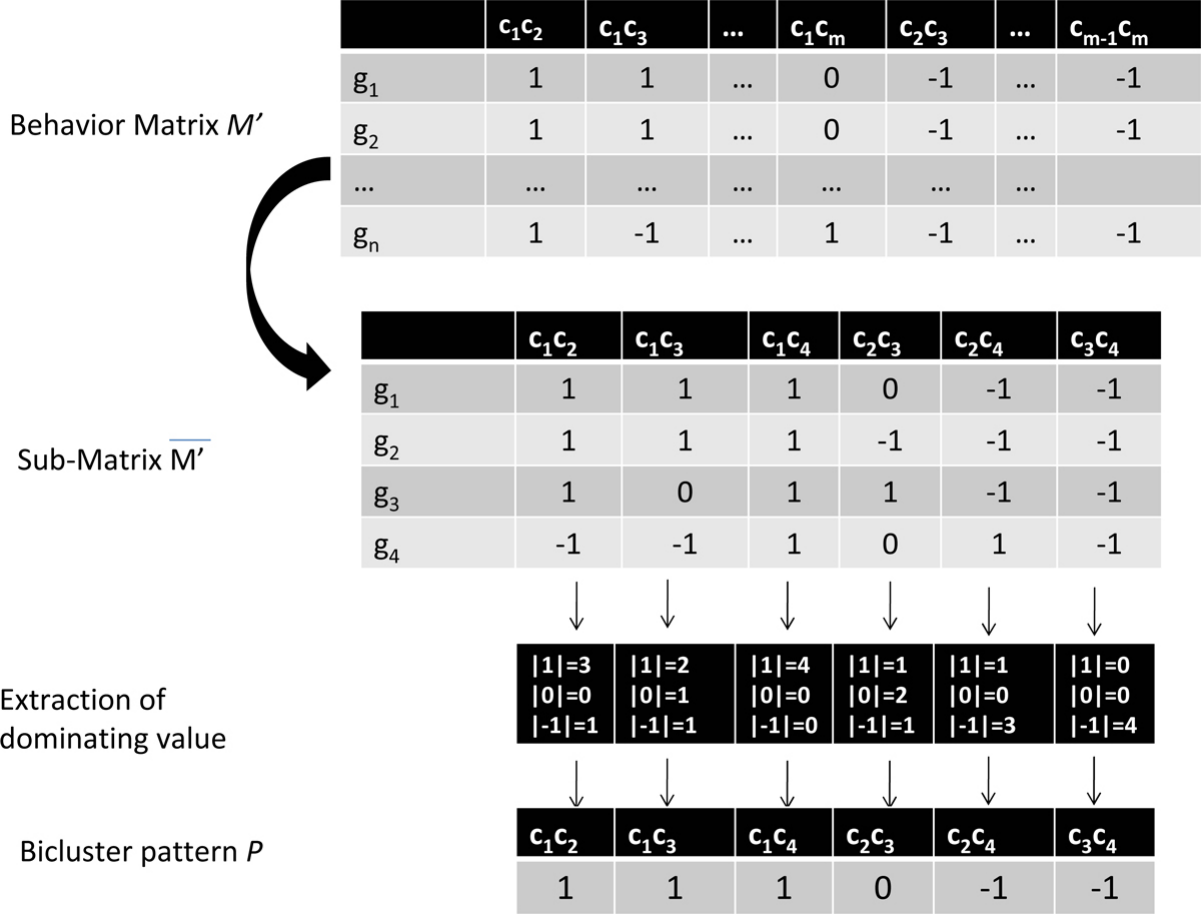
**Construction of bicluster pattern**.

Figure 1 shows an illustrative example. We can observe, by considering each row of *M'*, the trajectory (or behavior) pattern of each gene through all the combined conditions, i.e., up (1), down (-1) and no change (0), of all rows (genes) over combined columns (combined conditions). Similarly, the combinations of all the paired conditions give useful information since a bicluster may be composed of a subset of non contiguous conditions. Our PDNS algorithm uses *M' *to define its search space as well as its neighborhood that is critical for the search process.

### Pattern-driven neighborhood search for biclustering - general procedure

Our proposed PDNS method can be considered as an Iterated Local Search procedure [46]. It alternates between two basic components: a descent-based improvement procedure and a perturbation operator. PDNS uses the descent procedure to discover locally optimal solutions and the perturbation operator to displace the search to a new starting point in an unexplored search region.

The key originality of PDNS concerns the use of bicluster pattern both in its search space and neighborhood definition. The bicluster pattern is a characteristic representation of a bicluster. It is used to evaluate genes/conditions of bicluster. This representation is defined by the behavior matrix of the bicluster, i.e., the trajectory patterns of the genes under all combined conditions of the bicluster. This representation is important because it is well recognized that in microarray data, genes are considered to belong to the same cluster if they have similar trajectory patterns of expression levels [43-45,47].

Starting from an initial bicluster (call it current solution s), PDNS uses the descent strategy to explore the pattern-based neighborhood and moves to an improving neighboring solution at each iteration. By using the bicluster pattern, we define a set of rules which allow us to qualify the goodness (or badness) of a gene and condition. Using these rules (explained in a later section "Neighborhood and its exploration"), PDNS iteratively replaces within the current bicluster bad genes/conditions by good ones, thus progressively improves the quality of the bicluster under consideration. This iterative improvement procedure stops when the last bicluster attains a fixed quality threshold according to the ASR evaluation function (see next section) or when a fixed number *Y *of iterations is reached. At this point, PDNS triggers a perturbation phase by replacing randomly 10% of genes and conditions of the recorded best bicluster found so far. This perturbed bicluster is used as a new starting point for the next round of the descent search.

**Figure 2:**
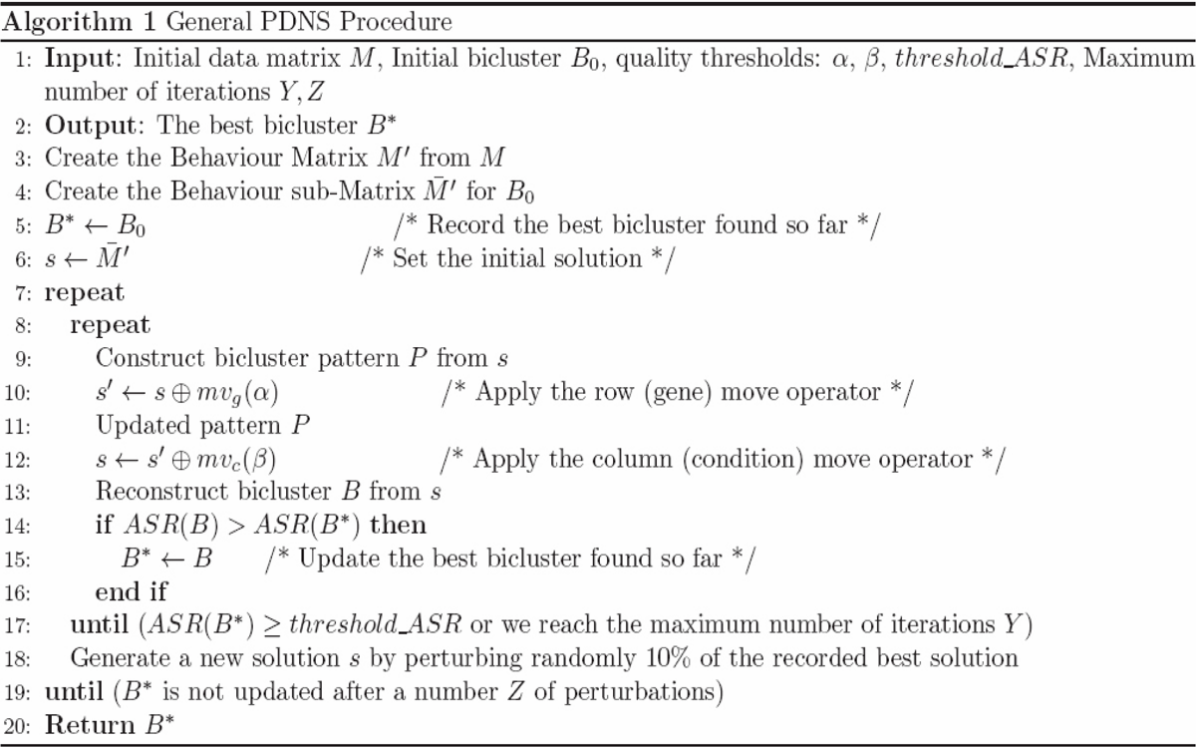
**General PDNS procedure**.

The whole PDNS algorithm stops when the best bicluster is not updated for a fixed number *Z *of perturbations. The general PDNS procedure is described in Figure 2. We describe in the following sections the ingredients of PDNS.

### The ASR evaluation function

Many functions exist for bicluster evaluation. One of the most popular evaluation functions is the *Mean Squared Residue *(MSR) [24]. It has been used by several biclustering algorithms [33,38,42,48-51]. However, MSR is deficient to assess correctly the quality of certain types of biclusters like multiplicative models [30,33,52,53].

In this paper, we use the Average Spearman's Rho (ASR) function which avoids the drawback of MSR [54]. Let *(I', J') *be a bicluster in a data matrix *M(I, J)*, the ASR evaluation function is then defined by:

(2) $$\text{ASR}(I^{\prime },J^{\prime })=2\;\text{max}\left\{\frac{\sum_{i\varepsilon I^{\prime }}\sum_{\begin{array}{c} j\varepsilon J^{\prime }\\ j\geq i+1 \end{array}}\rho_{ij}}{\left|I^{\prime }|(|I^{\prime }|-1\right)},\frac{\sum_{k\varepsilon J^{\prime }}\sum_{\begin{array}{c} l\varepsilon J^{\prime }\\ l\geq k+1 \end{array}}\rho_{kl}}{\left|J^{\prime }|(|J^{\prime }|-1\right)}\right\}$$

where *ρ_ij _*(*i ≠ j*) is the Spearman's rank correlation [55] associated with the row indices *i *and *j *in the bicluster (*I', J'*), *ρ_kl _*(*k ≠ l*) is the Spearman's rank correlation associated with the column indices *k *and *l *in the bicluster (*I', J'*). According to this definition, ASR(*I', J'*) ∈[-1..1].

A high (resp. low) ASR value, close to 1 (resp. close to -1), indicates that the genes/conditions of the bicluster are strongly (resp. weakly) correlated.

Let us notice that the existing evaluation functions can roughly be classified into two families: *numerical measures *and *qualitative measures*. *Numerical measures*, like *Pearson's correlation *or *Euclidean distance*, are easy to compute but they are quite sensitive toward outliers and noise. *Qualitative measures*, like measures that consider only ups, downs and no change of conditions, are very sensitive to precise the values of changes. As ASR is based on *Spearman's rank correlation *it can be considered as a good compromise between numerical and qualitative measures.

### Configuration representation

PDNS uses a solution representation based on the behavior matrix *M' *obtained from the preprocessing step described previously. More precisely, given a bicluster *B *= (*I'*, *J'*), we encode the bicluster by its behaviour matrix *s *= (*I',K*) which is the sub-matrix of *M' *including only the set of genes in *I' *and all the combinations of paired conditions in *J' *(see example of Figure 1). It is clear that *s *has the same rows as *B*, its number *K *of columns is equal to |*J'*|(|*J'*| - 1). In the rest of this paper, *s *is called a configuration (or solution). As it is shown below in Section "Neighborhood and its exploration", such a configuration representation enables the definition of dedicated move operators to improve progressively the quality of the generated biclusters.

### Initial solution

Our algorithm needs an initial bicluster to start its search. The initial bicluster can be provided by any means. For instance, this can be done randomly with a risk of starting with an initial solution of bad quality. A more interesting strategy is to employ a fast greedy algorithm to obtain rapidly a bicluster of reasonable quality. We use this strategy in this work and adopt two well-known algorithms: one is presented by Cheng and Church [24] and the other is called OPSM which is introduced in [29]. As explained above, each initial bicluster is encoded into its behavior matrix before being improved by PDNS.

### Neighborhood and its exploration

The neighborhood is one of the most critical elements of any local search algorithm. The neighborhood can be defined by a move operator. Given a solution *s*, let *mv *be the move operator that can be applied to *s*. Then each application of *mv *transforms *s *into a new solution *s'*. This is typically denoted by *s' *← *s *⊕*mv*.

In this paper, we devise two specially designed move operators operating respectively on rows (genes) and columns (combinations of pairwise conditions) of a given solution. Both operators are based on the general drop/add operation which removes some elements and adds new elements in the given solution. The critical issue here is the criterion that is employed to determine the elements to be removed and added. In our case, this decision is based on the "behavior pattern".

**Figure 3:**
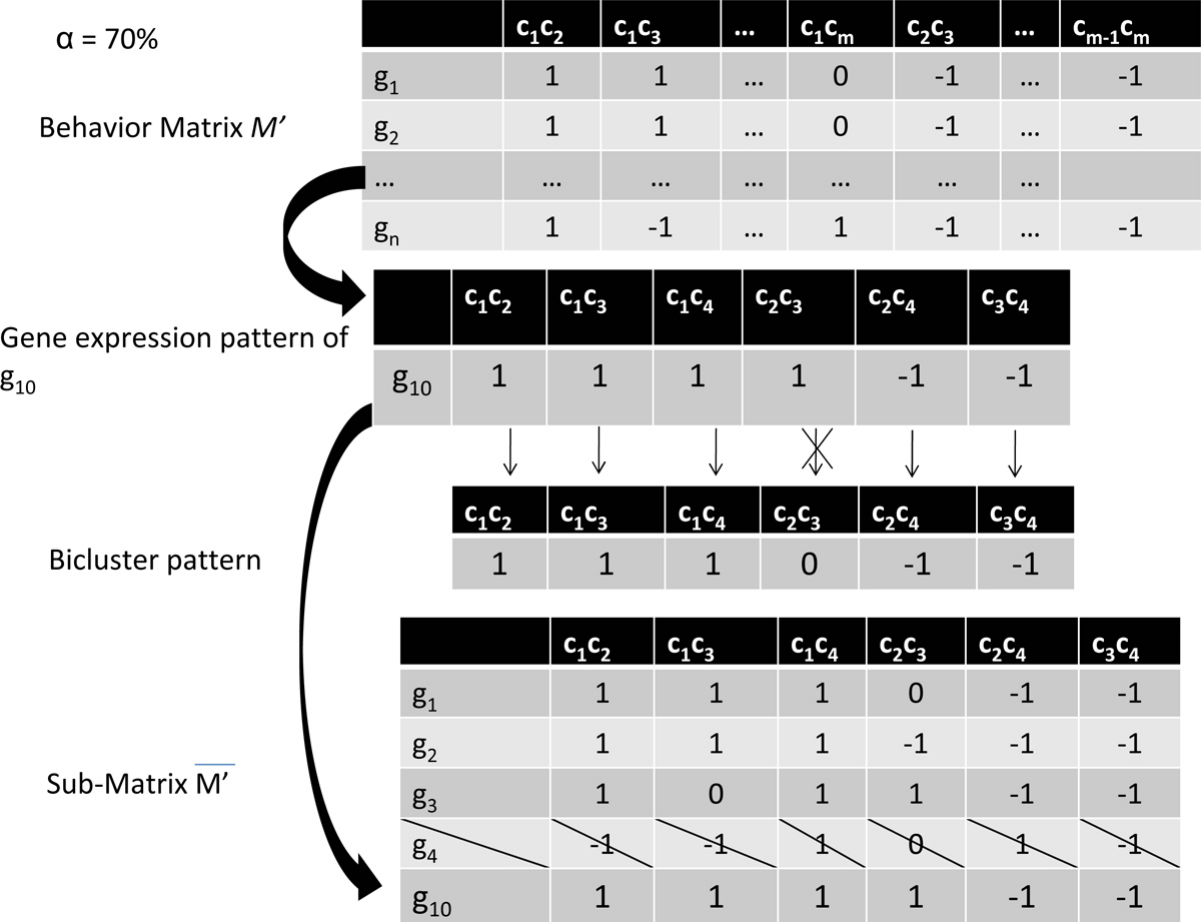
**Row move operator *mv_g_***. A bad gene (g_4_) is deleted since its quality (50%) is inferior to α = 70%; A good g_10 _is selected and added which has a quality (83%) superior to α = 70%.

Our first move operator, denoted by *mv_g_*, performs changes by removing a number of rows (genes) of the bicluster and adding other genes in order to obtain more coherent biclusters. Let *s *= (*I', K*) be a solution, we first extract from the behavior matrix *M' *the associated sub-matrix ${\bar{M}}^{\prime }$. Let *R *and *C *denote respectively the index set of rows and columns of ${\bar{M}}^{\prime }$. From ${\bar{M}}^{\prime }$ we build the bicluster pattern *P *of *s *which is defined by a vector indexed by *C*. *P*[*j*], *j *∈ *C*, takes the dominating value *k *∈ {1, 0, -1} such that *k *has the highest appearances in the column *i *of ${\bar{M}}^{\prime }$ (see example of Figure 3).

Now for each gene *g_i_*, *i *∈ *R *of the solution *s*, we define the quality of *g_i _*as the percentage of concordances between the behavior pattern of *g *and the behavior pattern *P *of bicluster *s*. Let α be a fixed quality threshold of genes. Let *D *denote the set of bad genes of *s *such that their quality does not reach the quality threshold fixed by α. Let *G *denote the set of good genes missing from *s *such that their quality surpasses the quality threshold α. Then our first move operator *mv_g _*removes from *s *all the bad genes of *D *and adds a number of genes selected from *G*.

Figure 3 shows an example where one bad gene (*g_4_*) is deleted and one good gene (*g_10_*) is added. *g_4 _*is bad because its behavior pattern has a low concordance with the bicluster behavior pattern (only 50% which is inferior than the quality threshold α = 70%). Similarly, *g_10 _*is good because its quality (83%) is higher than α. This replacement increases thus the coherence of the resulting bicluster. In the general case, the number of deleted gene may differ from the number of added genes. Notice that this move operator does not change the columns of the solution.

Our second move operator, denoted by *mv_c_*, performs changes by removing a number of columns (combined conditions) and adding other columns in order to obtain more coherent biclusters. Similar to the first move operator, *mv_c _*uses a quality threshold *β *for each column. The quality of each column is defined as the percentage of concordances between the column pattern and the value of this column in the bicluster pattern.

**Figure 4:**
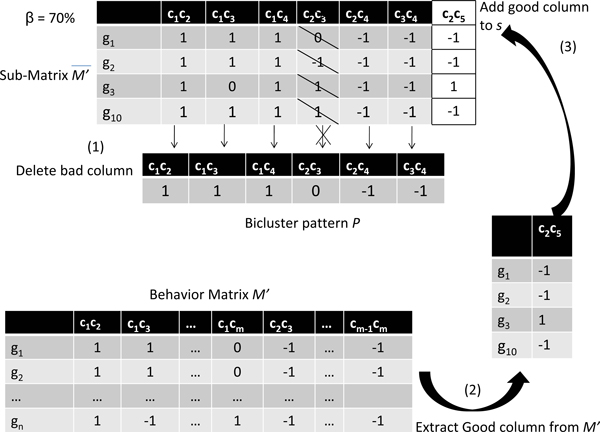
**Columns move operator *mv_c_***. Column c_2_c_3 _has a dominating value different to the column c_2_c_3 _in P and thus removed from s; c_2_c_5 _with a quality superior to β = 70% in the same subset of genes is selected and added into s.

Then, when our second move operator *mv_c _*detects a bad condition from the current bicluster, we test if the dominating value of each condition of the current bicluster has the same value with the corresponding value in the bicluster pattern. If it is different, this condition is considered bad (and removed from the current bicluster). To add a good condition from the current bicluster, we select a condition under the same subset of genes from the "behavior matrix" *M' *which has a dominating value higher than a fixed threshold *β*. Notice that this move operator does not change the rows of the solution (see example of Figure 4). In the general case, the number of deleted columns may differ from the number of added columns at each application of this move operator.

For a given solution, our PDNS algorithm applies these two move operators to reach a local optimum *s *(with an ASR value higher than the fixed *threshold_ASR *threshold). This local optimum solution *s *is composed of a group of genes and columns, each column representing the trajectory pattern of two conditions across the group of genes. Among the combinations of conditions in *s*, some conditions may be combined with only a few other conditions. These conditions are in fact insignificant conditions for the extracted bicluster. For this reason, during the decoding process (transforming *s *into a bicluster *B*), we retain only conditions which are combined with at least 50% other selected conditions. For instance, if we have *s *= {(*g_1_, g_2_, g_3_, g_4_*); (*c_1_c_2_, c_1_c_3_, c_1_c_4_, c_2_c_3_*)}, condition *c_4 _*will not be kept in the final bicluster because it is not combined at least with 50% of the other conditions, i.e., *c_2 _*and *c_3_*. The bicluster obtained is thus *B *= {(*g_1_, g_2_, g_3_, g_4_*); (*c_1_, c_2_, c_3_*)}.

## Results and discussion

### Experimental protocol

We perform statistical and biological validations of the obtained biclusters and we evaluate our PDNS algorithm against the results of some prominent biclustering algorithms used by the community, namely, CC [24], OPSM [29], ISA [56] and Bimax [57]. For these reference methods, we use *Biclustering Analysis Toolbox *(BicAT) which is a recent software platform for clustering-based data analysis that integrates all these biclustering algorithms [58]. We also compare our method with two additional methods (Samba [23] and RMSBE [27]).

For the experiments, we empirically fix α, *β *and *threshold_ASR *of the PDNS algorithm as follows. We experiment a number of combinations (typically several tens) and for each combination, we compute the *p*-values of the obtained biclusters. We pick the combination with the lowest *p*-value for the final experiment. For CC, OPSM, ISA and Bimax, the default values used in [27] are adopted for the Yeast Cell-Cycle dataset. For all the other experiments, we report the results of the compared algorithms from their original papers. The PDNS algorithm was implemented in Java and run on a PC Intel Core 2 Duo T6400 with 2.0GHz CPU and 3.5Gb RAM.

## Datasets and results

### Saccharomyces Cerevisiae dataset

The Saccharomyces Cerevisiae dataset (available at http://www.tik.ethz.ch/sop/bimax/) [59] contains the expression levels of 2993 genes under 173 experimental conditions. For this experiment, the parameters of PDNS are experimentally set as follows: α = 0.8, *β *= 0.8, *threshold_ASR *= 0.7, *Y *=100 and *Z*=50. The average running time of PDNS to improve a bicluster was about 4 minutes.

The results of PDNS are compared against the reported scores of RMSBE, Bimax, OPSM, ISA, Samba and CC from [27,57]. In order to evaluate the statistical significance of a bicluster, we determine whether the set of genes contained in the bicluster shows significant enrichment with respect to a specific *Gene Ontology *(GO). We use the webtool *FuncAssociate *(available at http://llama.mshri.on.ca/funcassociate/) [60] for this purpose. *FuncAssociate *computes the adjusted significance scores for each bicluster, i.e., adjusted *p*-values (*p *= 5%, 1%, 0.5%, 0.1% and 0.001%) which is the one-sided *p*-value of the association between attribute and query resulting from Fisher's Exact Test. The best biclusters have an adjusted *p*-value less than 0.001%.

**Figure 5:**
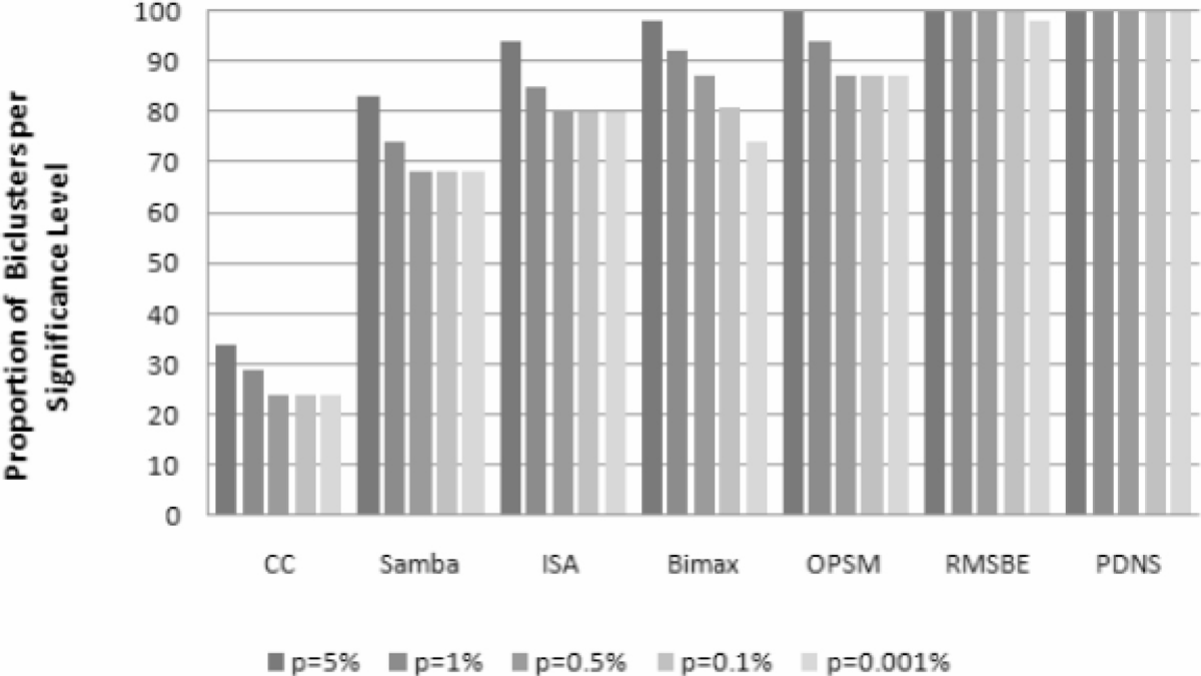
**Proportions of biclusters significantly enriched by GO on Saccharomyces Cerevisiae dataset**.

Figure 5 presents different significant scores *p *for each algorithm over the percentage of total extracted biclusters. On the one hand, PDNS and RMSBE seem to outperform other algorithms. PDNS (resp. RMSBE) results show that 100% (resp. 98%) of discovered biclusters are statistically significant with *p *< 0.001%. On the other hand, apart from CC, other algorithms have reasonably good performance. In particular, the best of the other compared algorithms, OPSM, 87% of its biclusters has *p *< 0.001%. CC under-performs because it is unable to find coherent biclusters and its lack of robustness against noise.

### Yeast Cell-Cycle dataset

The Yeast Cell-Cycle dataset (available at http://arep.med.harvard.edu/biclustering/) is described in [61]. This dataset is processed in [24] and publicly available from [62]. It contains the expression profiles of more than 6000 yeast genes measured at 17 conditions over two complete cell cycles. In our experiments we use 2884 genes selected by [24].

For this dataset, two criteria are used. First, we evaluate the statistical relevance of the extracted biclusters by computing the adjusted *p*-value like as for the Saccharomyces Cerevisiae dataset. Second, we identify the biological annotations for the obtained biclusters. For this experiment, the parameters α, *β*, *threshold_ASR*, *Y *and *Z *of PDNS are set as follows: α=0.5, *β *=0.7, *threshold_ASR *=0.5, *Y *=100 and *Z*=50. The average running time of PDNS to improve a bicluster was about 2 minutes.

#### Statistical relevance

**Figure 6:**
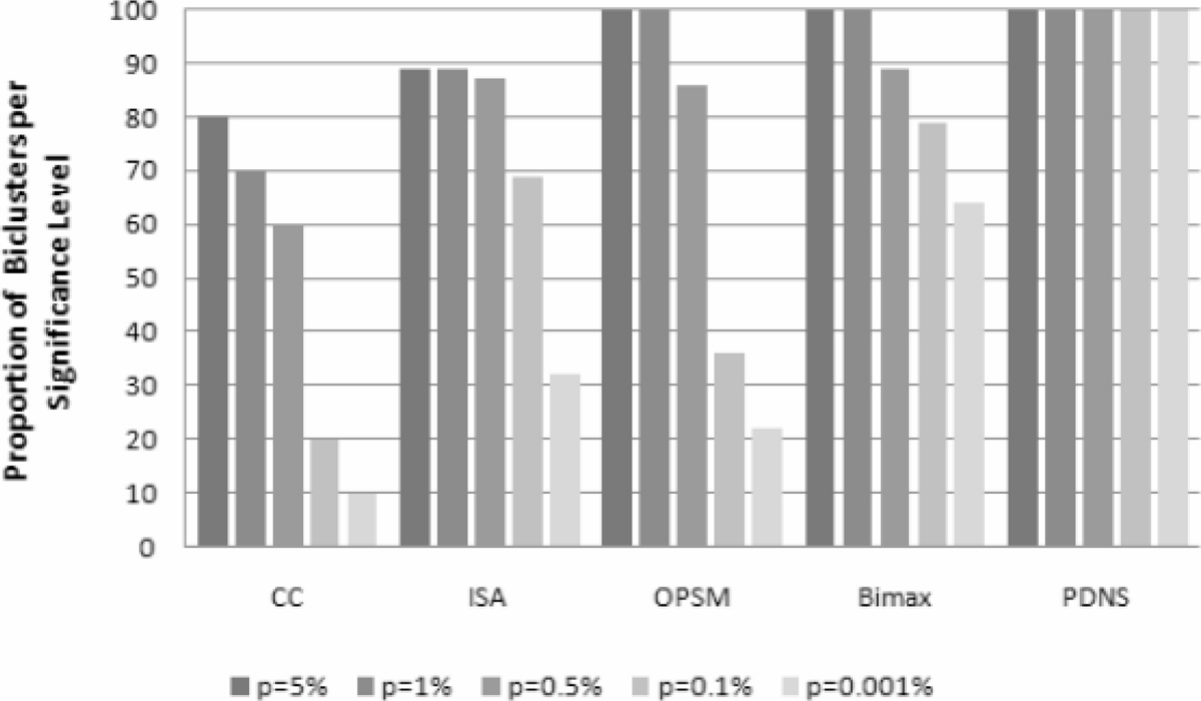
**Proportions of biclusters significantly enriched by GO on Yeast Cell-Cycle dataset**.

To evaluate the statistical relevance of PDNS, we use again the *p*-values and apply the web-tool *FuncAssociate *[60]. The results of PDNS are compared against CC, ISA, Bimax and OPSM. Figure 6 shows, for each significant score *p *(*p *= 5%, 1%, 0.5%, 0.1% and 0.001%) and for each compared algorithm, the percentage of the statistically significant biclusters extracted by the algorithm with the indicated *p*-value. We observe that PDNS outperforms the other algorithms on this dataset. 100% of discovered biclusters of PDNS are statistically significant with *p *< 0.001%. However, the best of the compared algorithm (Bimax) has only a percentage of 64% for *p *< 0.001%.

#### Analysis of biological annotation enrichment of biclusters

To evaluate the biological significance of the obtained biclusters in terms of the associated biological processes, molecular functions and cellular components respectively, we use the Gene Ontology (GO) term finder *GOTermFinder *(available at http://db.yeastgenome.org/cgi-bin/GO/goTermFinder). Indeed, the GO project provides a controlled vocabulary to describe gene and gene product attributes in any organism, and it is a collaborative effort to address the need for consistent descriptions of gene products in different databases (cited from http://www.geneontology.org). *GOTermFinder *can find the significant shared GO terms for genes within the same bicluster.

Table 1 and Table 2 report the top GO terms shared by the biclusters of CC (*id2_CC_*, *id9_CC_*) and OPSM (*id7_OPSM_*, *id10_OPSM_*), and their improvement by PDNS (*id2_PDNS_*, *id9_PDNS_*, *id7_PDNS_*, *id10_PDNS_*), in terms of biological process, molecular function and cellular component. For each GO, we list only the most significant shared term with the smallest *p*-value.

**Table 1:** Most significant shared GO terms (process, function, component) of CC and PDNS for biclusters on Yeast Cell-Cycle dataset

**Bic**.	**Algo**.	Biological process	Molecular function	Cellular component
*id9_CC_* *id9_PDNS_*	CC PDNS	unknown glutamate biosynthetic process (10.2%, 8.62e-08)	unknown isocitrate dehydrogenase (NAD+) activity (18.6%, 0.00300)	unknown mitochondrion part (48.3%, 5.19e-07)

*id2_CC_* *id2_PDNS_*	CC PDNS	translation (46.6%, 1.72e-22) translation (58.1%, 8.71e-37)	structural constituent of ribosome (38.8%, 1.05e-36) structural constituent of ribosome (51.3%, 4.48e-59)	cytosolic ribosome (38.8%, 1.10e-41) cytosolic ribosome (53.00%, 5.97e-70)

**Table 2:** Most significant shared GO terms (process, function, component) of OPSM and PDNS for biclusters on Yeast Cell-Cycle dataset

**Bic**.	**Algo**.	Biological process	Molecular function	Cellular component
*id7_OPSM_* *id7_PDNS_*	OPSM PDNS	unknown ribosome biogenesis (32.1%, 2.02e-07)	unknown snoRNA binding (5.3%, 5.84e-06)	unknown nucleolus (32.1%, 6.22e-10)

*id10_OPSM_* *id10_PDNS_*	OPSM PDNS	sister chromatid segregation (24.7%, 0.00337) nucleic acid metabolic process (34.0%, 2.45e-11)	unknown phosphatase regulator activity (1.7%, 0.00041)	spindle (14.1%, 0.00196) nucleus (44.8%, 3.46e-15)

For the bicluster labeled *id9_PDNS _*(Table 1), the genes *YCR005C, YHR037W, YLR304C, YNL037C, YNR001C *and *YOR136W *are together involved in the glutamate biosynthetic process. Each GO term is associated with a tuple, for example glutamate biosynthetic process (10.2%, 8.62e-08) indicates the cluster frequency and the statistical significance. The cluster frequency (10.2%) shows that out of 59 genes in the first bicluster 6 genes take part to this process, and the statistical significance is provided by a *p*-value of 8.62e-08. Furthermore, PDNS can improve all the biclusters of CC (resp. OPSM) and find biologically meaningful biclusters.

For the worst (resp. the best) biclusters obtained from CC, i.e, *id9_CC _*(resp. *id2_CC_*) and OPSM, i.e., *id7_OPSM _*(resp. *id10_OPSM_*), we verify whether the PDNS algorithm can improve these biclusters to obtain biclusters of more relevant biological significance. We observe that PDNS does improve the worst and the best biclusters of CC and OPSM. For the worst biclusters which have no biological significant ("unknown"), i.e., *id9_CC _*and *id7_OPSM_*, the improved biclusters obtained by PDNS (*id9_PDNS _*and *id7_PDNS_*) tend to be more statistically and biologically significant. Indeed, when a bicluster has a bad quality, PDNS can improve it by replacing the bad genes/conditions by the good ones. For the best biclusters, i.e., *id2_CC _*and *id10_OPSM_*, PDNS can also improve them (*id2_PDNS _*and *id10_PDNS_*) by improving the respective *p*-value.

## Conclusions

We have presented the pattern-driven neighborhood search for the biclustering problem of microarray data. PDNS alternates between a descent-based intensification phase and a perturbation phase. By using a behavior matrix representation of solutions, the descent search procedure is guided by a pattern-based neighbourhood which is defined by two move operators. These operators change respectively the rows and columns of the current solution according to the pattern information related to each row and each column of the current solution as well as the initial matrix. Perturbation is realized by changing randomly a percentage of rows and columns of the best recorded solution (an option would be to constraint the changes to some critical rows and columns).

The proposed algorithm has been assessed using two well-known microarray datasets (Yeast Cell-Cycle and Saccharomyces Cerevisiae). The experimental study showed competitive results of PDNS in comparison with other popular biclustering algorithms by providing statistically and biologically significant biclusters. PDNS is a computationally effective method and can also be used to improve biclusters obtained by other methods.

## Competing interests

The authors declare that they have no competing interests.

## Authors' contributions

WA carried out the implementation of the proposed idea, performed the statistical and biological experiments using *FuncAssociate *and *GoTermFinder*, and wrote the draft manuscript. JKH supervised the project and co-wrote the manuscript. ME participated in the correction of the final manuscript. All authors read and approved the final manuscript.
